# Frequency-Dependent Amplitude Alterations of Resting-State Spontaneous Fluctuations in Late-Onset Depression

**DOI:** 10.1155/2015/505479

**Published:** 2015-02-01

**Authors:** Yingying Yue, Xize Jia, Zhenghua Hou, Yufeng Zang, Yonggui Yuan

**Affiliations:** ^1^Department of Psychosomatics and Psychiatry, Affiliated Zhongda Hospital of Southeast University, Medical School of Southeast University, No. 87, Dingjiaqiao Road, Nanjing 210009, China; ^2^Center for Cognition and Brain Disorders and the Affiliated Hospital, Hangzhou Normal University, Hangzhou, Zhejiang 310000, China; ^3^Zhejiang Key Laboratory for Research in Assessment of Cognitive Impairments, Hangzhou, Zhejiang 310000, China; ^4^Department of Psychiatry, The 4th People's Hospital of Wuhu City, Wuhu 241000, China

## Abstract

There is limited amplitude of low-frequency fluctuation (ALFF) of resting-state functional magnetic resonance imaging (fMRI) studies in late-onset depression (LOD) but reported different results. This may be due to the impact of different frequency bands. In this study, we examined the ALFF in five different frequency bands (slow-6: 0–0.01 Hz; slow-5: 0.01–0.027 Hz; slow-4: 0.027–0.073 Hz; slow-3: 0.073–0.167 Hz, and slow-2: 0.167–0.25 Hz) within the whole brain during resting-state fMRI in 16 LOD patients and 16 normal control (NC) subjects. The ALFF of primary effect of disease was widely distributed over left cerebellum anterior lobe, left cerebellum posterior lobe, left middle orbitofrontal gyrus, left superior occipital, and right superior parietal, while the interaction effect of disease and frequency was distributed over right superior frontal gyrus. Further relationship analysis findings suggest these abnormal ALFF may relate to cognitive dysfunction of LOD. Therefore, our data show that LOD patients have widespread abnormalities in intrinsic brain activity, which is dependent on the frequency band, and suggest that future studies should take the frequency bands into account when measuring intrinsic brain activity.

## 1. Introduction

Late-onset depression (LOD) is an important public health problem due to its high prevalence of 5%–10% and is characterized by increased anhedonia, apathy, and cognitive impairment, so it is often referred to as either a pseudodementia syndrome or a prodrome of dementia [1–4]. Moreover, longitudinal follow-up studies showed that remitted geriatric depression (RGD) still showed poorer cognitive function compared to healthy controls, even after the remission of mood symptoms [5, 6]. Understanding the pathophysiology of LOD is a clear goal in achieving further advances in the therapy of LOD and prevents converting to dementia.

In recent years, neuroimaging studies have greatly advanced our understanding of the pathogenesis of LOD. Resting-state functional magnetic resonance imaging (rs-fMRI) is an effective method to investigate the pathogenesis of MDD since Biswal and his colleagues first reported that spontaneous blood oxygen level-dependent (BOLD) low-frequency (0.01–0.08 Hz) fluctuations were physiologically meaningful and were related to neural spontaneous activity [7]. In rs-fMRI, regional homogeneity (ReHo) and amplitude of low-frequency fluctuation (ALFF), which reflects the temporal changes in neural activity in brain regions, and functional connectivity (FC) analysis, which measures the correlation coefficients of all brain areas with a predefined region, have often been used. Previous study found lower ReHo values in right precuneus, bilateral frontal gyrus and higher ReHo values in left superior temporal gyrus, left cerebellum [8]. In addition, this frontal gyrus showed reduced FC with the left amygdala [9]. Diffusion tensor imaging observed lower fractional anisotropy values in frontolimbic areas (superior frontal gyrus and parahippocampal gyrus) in LOD patients [10, 11]. From intraregional perspectives, amplitude of low-frequency fluctuation (ALFF) is the approach for analyzing rs-fMRI data which use voxel-based analysis and focus on regions of spontaneous activity in the whole brain, which is an index reflecting directly the intensity of spontaneous neural activity within a specific frequency range without filtering at the baseline state [12, 13]. ALFF were widely used in the related study of diagnosis and treatment of depression including major depressive disorder (MDD), early onset depression (EOD), and late-onset depression (LOD). The ALFF in MDD patients was significantly increased in many brain regains including the right precentral gyrus, right inferior temporal gyrus, and bilateral anterior and posterior lobes of the cerebellum but was decreased in the widely cortical regions (prefrontal-temporal-parietal lobe) [14]. In addition, Guo et al. [15] demonstrated that LOD had lower ALFF in bilateral precuneus, superior medial frontal gyrus and superior frontal gyrus, and higher ALFF in left brainstem and left superior temporal gyrus compared to EOD patients. Further ROC analysis suggested that the mean ALFF values in the bilateral superior frontal gyrus and left superior temporal gyrus could serve as markers to separate patients with EOD from individuals with LOD.

To date, most rs-fMRI studies have examined spontaneous low-frequency oscillations (LFO) activities at a specific frequency band of 0.01–0.08 Hz because the frequency band was thought to be linked to neuronal fluctuations [7, 16, 17]. However ALFF is effective in detecting LFO; it may also include frequency over 0.1 Hz. Nonetheless, previous study has found that neuronal oscillations are distributed linearly on the natural logarithmic scale and independent frequency bands which are generated by distinct oscillators with specific properties and physiological functions [18, 19]. Moreover, neighboring frequency bands within the same neuronal network may compete or interact with each other [20]. Zuo et al. [21] have shown that low-frequency amplitudes in the slow-4 band were higher than those in the slow-5 in the basal ganglia, thalamus, precuneus, and so on by decomposing into four distinct frequency bands (slow-5 (0.01–0.027 Hz), slow-4 (0.027–0.073 Hz), slow-3 (0.073–0.198 Hz), and slow-2 (0.198–0.25 Hz)). One possibility is that the amplitudes of LFO are frequency-dependent. However, it remains largely unknown whether LOD patients show abnormal frequency-dependent changes in the ALFF.

In the present study, we applied ALFF to examine the amplitude of LFO in LOD and NC groups at different frequency bands (slow-6: 0–0.01 Hz, slow-5: 0.01–0.027 Hz, slow-4: 0.027–0.073 Hz, slow-3: 0.073–0.167 Hz, slow-2: 0.167–0.25 Hz). Then the relationship between regions identified as showing significant difference between the two groups (LOD and NC groups) and the severity of depression and cognitive function were explored.

## 2. Materials and Methods

### 2.1. Participants

This study was approved by the Medical Ethics Committee for Clinical Research of Zhongda Hospital Affiliated to Southeast University. All patients and healthy controls gave their written informed consent to participate in the study. A total of 16 LOD inpatients and 16 age- and sex-matched healthy controls were recruited. The inclusion criteria for patient group included (1) meeting the diagnostic criteria for MDD using a Structured Clinical Interview by two trained senior psychiatrists (Zhenghua Hou and Yonggui Yuan) according to the Diagnostic Statistical Manual of Mental Disorder, Fourth Edition (DSM-IV); (2) the patients were first onset after 60 years and medication-naive; (3) Hamilton Depression Rating Scale (HDRS-17) score greater than 17; (4) being right-handed; (5) being free of other major psychiatric disorders, cerebrovascular diseases, and severe physical illnesses; and (6) no contraindications to MRI scanning. The inclusion criteria for NC participants were similar to LOD patients except fulfilling the diagnostic criteria for MDD and HDRS-17 score was less than 8. Diagnostic evaluations were carefully conducted on all participants, which included a clinical interview, a focused neurological and mental status exam, and a demographic inventory.

### 2.2. The Evaluation of Depression and Neuropsychological Measurements

All subjects underwent diagnostic evaluations, including HDRS-17; the Hamilton Anxiety Rating Scale (HARS); and cognitive function testing with a neuropsychological battery that consisted of the Mini Mental State Examination (MMSE), the Auditory Verbal Learning Test (AVLT-) delayed recall, the Digit Span Test (DST-forward and backward), the Symbol Digit Modalities Test (SDMT), the Verbal fluency test (VFT-animal and verb), and the Trail Making Test (TMT-A and B) [22]. We merged the measurement of similar cognitive domain and the process is as follows. First, all scale scores were transformed to standard *Z* value in order to avoid the influence of the different measurement units. Second, the scale scores of representing the same domain were added up. This set of neuropsychological tests was grouped into the following domains: overall cognitive function (MMSE), memory function (AVLT-delayed recall), language (VFT), executive function (TMT-B), processing speed (SDMT, TMT-A), and attention function (DST).

### 2.3. Image Acquisition and Processing

The subjects were scanned using a Siemens 3.0 Tesla scanner and a standard head coil. Subjects lay supine with the head snugly fixed by a belt and foam pads to minimize head motion. A gradient-recalled echo-planar imaging (GRE-EPI) pulse sequence was set up to acquire resting-state images. Scan parameters were as follows: 31 axial slices, repetition time = 2000 ms; echo time = 30 ms; flip angle = 90°; acquisition matrix = 64 × 64; field of view = 240 × 240 mm^2^; thickness = 4.0 mm; gap = 0 mm; and 3.75 × 3.75 mm^2^ in-plane resolution parallel to the anterior commissure-posterior commissure line. This acquisition sequence generated 140 volumes in 4 min and 40 s. All subjects were instructed to close their eyes and not to think of specific things during scanning.

### 2.4. Functional Image Processing

The preprocessing of rs-fMRI images was performed using DPARSF [23] (http://www.restfmri.net) and REST [24] (http://www.restfmri.net). We performed the following preprocessing steps on the rs-fMRI images: (1) discarding of the first 10 volumes of functional images for signal stabilization and adaptation to inherent scanner noise; (2) slice timing; (3) head motion correction (participants with head motion of more than 2.5 mm of maximum displacement in any direction (*x*, *y*, or *z*) or 2.5 degrees of angular motion were excluded from the present study); (4) spatial normalization and then resampling of the functional images to 3 mm isotropic voxels; (5) spatial smoothing with an isotropic Gaussian kernel with a FWHM of 4 mm; (6) removing the linear trend within the time series.

ALFF was calculated by REST software with five different frequency ranges (slow-6: 0–0.01 Hz, slow-5: 0.01–0.027 Hz, slow-4: 0.027–0.073 Hz, slow-3: 0.073–0.167 Hz, and slow-2: 0.167–0.25 Hz) separately. The procedure for calculating ALFF was briefly described as follows. For a given voxel, the time series was first converted to the frequency domain using a Fast Fourier Transform. The square root of the power spectrum was computed and then averaged across a predefined frequency interval. This averaged square root was termed the ALFF at the given voxel [8]. Then, ALFF was standardized by dividing the whole brain voxel average ALFF, which measures the absolute strength or intensity of spontaneous LFO.

### 2.5. Statistical Analysis

To explore the ALFF differences between two groups and different frequency, the effects of disease and frequency were examined by two-way analysis of variance (ANOVA) using AFNI software (http://afni.nimh.nih.gov/afni) within a gray matter mask. One of the factors in the ANOVA accounted for disease, with two levels (LOD and NC), and the other factor accounted for the different frequency by ALFF, with five levels (slow 6–2). All the statistical maps were corrected for multiple comparisons to a significant level of *P* < 0.05 (bilateral) by combining the individual voxel *P* value <0.05 with cluster size >2079 mm^3^ based on using Monte Carlo simulations [25].

Two independent sample *t*-tests and Chi-squared tests were used to compare demographic performance (statistical significance was set at *P* < 0.05). Mann-Whitney *U* test was used to compare cognitive function.

Further correlative analysis between ALFF value of significant different brain area and neuropsychological performance was then performed on the LOD groups by extracting of the most significant different frequency between groups. These analyses were performed using the REST extract ROI Series [24] (http://www.restfmri.net). Then, Spearman's correlative analyses were performed to examine relationships between abnormal ALFF and standardized neuropsychological performance scores using SPSS 18.0 software (SPSS, Inc., Chicago, IL).

## 3. Results

### 3.1. Neuropsychological Results

There are no differences of age, gender, and education levels between LOD and NC groups. Compared with NC, LOD patients displayed comprehensive deficits in cognitive performance, including language, attention, and memory functions. However, the total cognitive function, processing speed, and executive function were similar for the two groups (see Table 1).

### 3.2. ALFF Results

**Figure 1:**
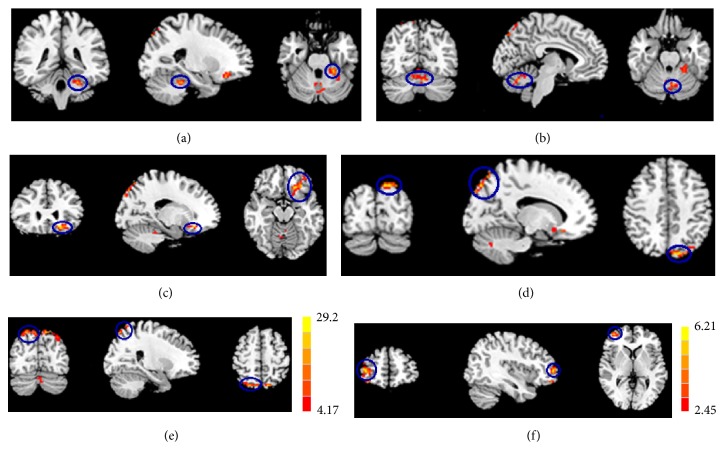
Maps of the main effect of disease between LOD and NC groups. The ALFF of primary effect of disease was widely distributed over left cerebellum anterior lobe, left cerebellum posterior lobe, left middle orbitofrontal gyrus, left superior occipital, and right superior parietal. (a) Cluster 1 mainly includes left cerebellum anterior lobe; (b) cluster 2 includes left cerebellum posterior lobe; (c) cluster 3 includes left middle orbitofrontal gyrus; (d) cluster 4 mainly includes left superior occipital gyrus and left precuneus; (e) cluster 5 mainly includes right superior parietal gyrus and right precuneus. (f) The interaction of disease and frequency was distributed over right superior frontal gyrus. A single voxel threshold of the map resulting from the *F*-test was set at a *P* < 0.05, and a minimum cluster size of 2079 mm^3^ was used to correct by Monte Carlo simulation.

**Figure 2:**
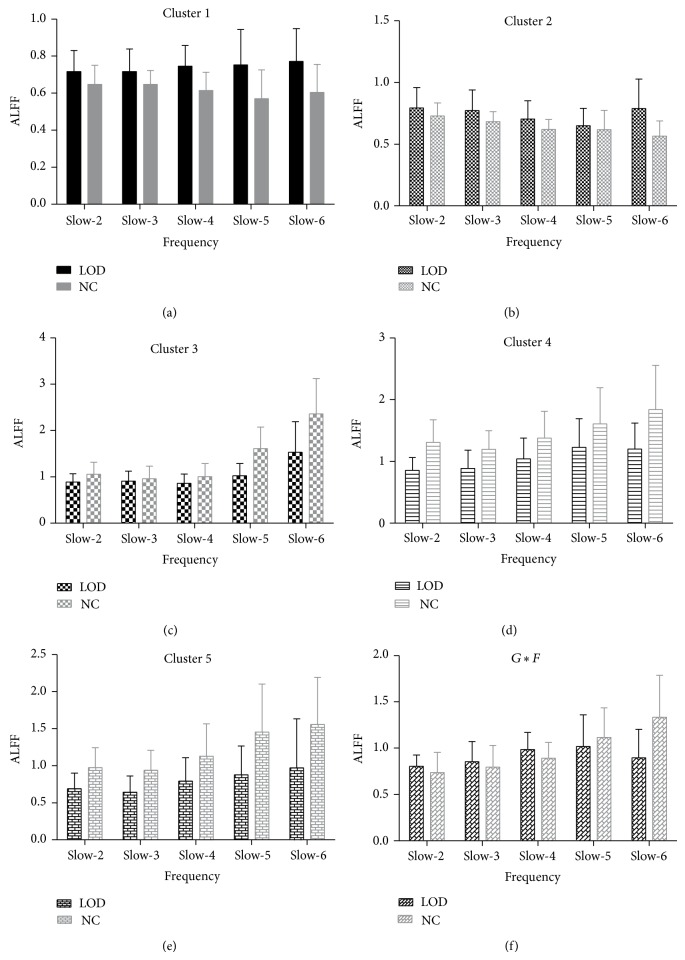
The bar graph showed the significant clusters of abnormal ALFF between LOD and NC groups with different frequency bands (slow-6: 0–0.01 Hz, slow-5: 0.01–0.027 Hz, slow-4: 0.027–0.073 Hz, slow-3: 0.073–0.167 Hz, and slow-2: 0.167–0.25 Hz). Cluster 1 mainly includes left cerebellum anterior lobe, cluster 2 includes left cerebellum posterior lobe, cluster 3 includes left middle orbitofrontal gyrus, cluster 4 mainly includes left superior occipital gyrus and left precuneus, and cluster 5 mainly includes right superior parietal gyrus and right precuneus. *G*∗*F*: the interaction of group and frequency was distributed over right superior frontal gyrus.

The ALFF of primary effect of disease was widely distributed over left cerebellum anterior lobe, left cerebellum posterior lobe, left middle orbitofrontal gyrus, left superior occipital, and right superior parietal (cluster 1 mainly includes left cerebellum anterior lobe, cluster 2 includes left cerebellum posterior lobe, cluster 3 includes left middle orbitofrontal gyrus, cluster 4 mainly includes left superior occipital gyrus and left precuneus, and cluster 5 mainly includes right superior parietal gyrus and right precuneus; see Table 2 and Figures 1 and 2). Statistical *F*-tests for the interaction effect of both factors were performed. A single voxel threshold of the map resulting from the *F*-test was set at a *P* < 0.05, and a minimum cluster size of 2079 mm^3^ was used to correct for multiple comparisons, determined by Monte Carlo simulation. The interaction of disease and frequency was distributed over right superior frontal gyrus.

### 3.3. Relationships between Abnormal ALFF and Neuropsychological Assessments

**Figure 3:**
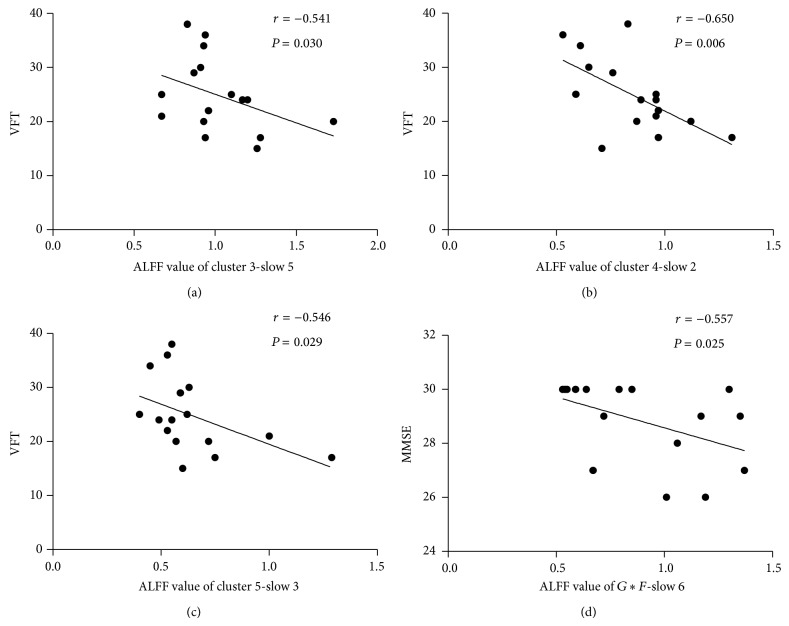
Abnormal clusters of ALFF distracted by the largest contribution had significant correlation to cognitive dysfunction in LOD patients. The left middle orbitofrontal gyrus with the 0.01–0.027 Hz (cluster 3-slow 5), left superior occipital gyrus with 0.167–0.25 Hz (cluster 4-slow 2), and right superior parietal gyrus with 0.073–0.167 Hz (cluster 5-slow 3) were, respectively, negatively related to VFT (*r* = −0.541, *P* = 0.030; *r* = −0.650, *P* = 0.006; *r* = −0.546, *P* = 0.029), while the cluster-slow 6 of interaction of disease and frequency had negative correlations with MMSE (*r* = −0.557, *P* = 0.025). MMSE: Mini Mental State Examination, VFT: Verbal fluency test. *r* = Spearman's correlation coefficient.

The correlation between abnormal ALFF and cognitive function was calculated in LOD group. The ALFF of abnormal clusters was distracted by the largest contribution. In the LOD patients, the left middle orbitofrontal gyrus with the 0.01–0.027 Hz (cluster 3-slow 5), left superior occipital gyrus with 0.167–0.25 Hz (cluster 4-slow 2), and right superior parietal gyrus with 0.073–0.167 Hz (cluster 5-slow 3) were, respectively, negatively related to VFT (*r* = −0.541, *P* = 0.030; *r* = −0.650, *P* = 0.006; *r* = −0.546, *P* = 0.029), while the right superior frontal gyrus with 0–0.01 Hz of interaction of disease and frequency had negative correlations with MMSE (*r* = −0.557, *P* = 0.025; see Figure 3).

## 4. Discussion

To our knowledge, this is the first study investigating the different frequency bands amplitude of LFO in LOD and NC groups. The principal finding was that the wide brain regions including left cerebellum anterior lobe, left cerebellum posterior lobe, left middle orbitofrontal gyrus, left superior occipital, and right superior parietal might contribute to the pathogenesis on LOD in the whole frequency bands while the interaction of disease and frequency was distributed over right superior frontal gyrus. The other finding is that the abnormal brain regions between two groups were associated with cognitive impairment, but not with the severity of depression.

Biswal is the first who discovered the ALFF reflected the local spontaneous activity of resting state. In 2004, electrophysiological studies indicate low-frequency oscillation may be due to the generation of spontaneous neuronal activity which generated its rhythmic activity patterns through information exchange with neighboring brain regions and had a physiological sense, so it can be used as brain response characteristics. Although the origins and functional significances of different frequency bands remain unclear, previous studies have suggested that lower frequency oscillations allow for an integration of large neuronal networks whereas higher frequency oscillations are confined to small neuronal space [18]. Because most neuronal connections are local, the period of oscillation is constrained by the size of neuronal pool engaged in a given cycle [18, 26]. The cortical regions have relatively large sizes and may contribute to the long distance connections in very large networks. In contrast, subcortical regions phylogenetically older may mainly contribute to fast local events, which are modulated by widespread slow oscillations [18]. Before, frequency-dependent changes in the ALFF have been found in amnestic mild cognitive impairment and schizophrenia [27, 28]. Therefore, the network mechanisms and the disease phenotype of these different LFO bands are interesting topics.

However, to date, most rs-fMRI studies have examined spontaneous LFO activities at a type frequency band of 0.01–0.08 Hz which was thought to be mainly linked to neuronal fluctuations; no study explored the different frequency ALFF in LOD patients. The major finding of present study is that increased ALFF were found in the left cerebellum lobe and decreased ALFF in the left middle orbitofrontal gyrus, left superior occipital, and right superior parietal of LOD patients. Previous study demonstrated that LOD had lower ALFF in bilateral superior frontal gyrus and higher ALFF in left superior temporal gyrus. Further ROC analysis suggested that the mean ALFF values in the bilateral superior frontal gyrus and left superior temporal gyrus could serve as markers to separate patients with EOD from individuals with LOD [15]. In the positron emission tomography (PET) study discovered decreased relative metabolism in the frontal lobe, cingulate and parietal regions in both hemispheres in LOD patients [29]. The Cohe-ReHo, an advanced accessing regional spontaneous neural activity analytical method, showed significantly decreased value in left dorsolateral prefrontal cortex, bilateral medial prefrontal cortex, and right precuneus, while it showed significant increased ReHo value in left cerebellum posterior lobe [30]. The bilateral precuneus is viewed to be an important component of default mode network (DMN) which associated with self-related cognitive processing, such as autobiographical memory [31, 32]. Together with the above-mentioned findings, we speculate that the hypoactivity of the precuneus and parietal-occipital lobe may contribute to the disorder of DMN, thereby leading to the MDD-related pathopsychological characteristic. Our results adding to previous literature suggest that depression patients had abnormal amplitude over the frontal, parietal, occipital, and cerebellum regions.

In these regions, increased ALFF value of bilateral cerebellum regions was an interesting finding. In contrast to the many neuroimaging studies investigating cerebral cortex, little attention has been paid to the alterations in the cerebellum traditionally thought as a region coordinates motor behavior [33], but the notion that this region involved in emotional control played an important role in the perception of emotional stimuli has recently gained popularity [34, 35]. The cerebellum is anatomically connected with brainstem reticular nuclei and limbic regions and receives projections from the caudal and rostral anterior cingulate via the pons [36–38]. Thus, these connections may provide an anatomical foundation for the cerebellum's emotional role. The rs-fMRI study exhibited altered neural response in cerebellum activity. The increased cerebellar activity at resting state may be a disease state phenomenon but not a compensatory response to the dysfunction of other brain regions or neural circuit in depressive patients [39, 40].

The right superior frontal gyrus was interaction of disease and frequency in this frequency-specific study. In addition, our previous study found that superior frontal gyrus had decreased ReHo in remitted geriatric depression [41]. The frontal lobe is one of the most consistently identified regions as associated with depressive disorder [42, 43]. Cognitive bias towards negative information and away from positive information contributes to the maintenance of depressed mood according to Beck's cognitive model [44]. Also the dorsal frontal gyrus plays an essential role in the control of information processing and decision making by inhibiting irrelevant neural activity. Reduced metabolism of the frontal gyrus observed through different methods and strategies might be related to increased effort to effectively inhibit subcortical brain activity [45–47]. This brain area is influenced not only by disease but also by frequency in our study suggesting that frequency related factors should be considered in fMRI study to get reliable results. Above findings further suggest that frontal gyrus dysfunction may underlie vulnerability to depression and perhaps participate in impaired emotional and cognitive regulation in LOD patients.

Our correlative analyses conducted between the changes of imaging indicators and neuropsychological performance in the LOD group showed that no significant correlations were found between ALFF measurements in any region and illness severity, but involved in total cognitive function and language function. This phenomenon intuitively suggests that the ALFF cannot be used as a quantitative marker for the assessment of depressive severity at this stage, although these indicators are helpful for the localization of functionally aberrant regions. LOD is a mood disorder and is generally characterized as depressed mood or loss of interest or pleasure, whereas the increasing recognition found it is also a cognitive disorder [48]. Comorbid depression and cognitive impairment increase the rate of adverse outcomes for physical health, functional status, and mortality [49]. Our result was consistent with previous studies that LOD patients showed multidimensional cognitive impairment except total cognitive function, processing speed, and executive dysfunction. Above cognitive function may operate through frontosubcortical disconnection, white matter lesions including of high-intensity in deep white matter and disruption of white matter integrity as well as other brain structural impairment [50, 51]. The present study finding abnormal ALFF in frontal-parietal-occipital regions and cerebellum reflects functional changes that may be before structural changes, which can be comprehended that the changes of local brain activity induced some cognitive disorder to a certain extent.

This present study is an exploring study and several limitations should be considered. Firstly, this was a cross-sectional study with a relatively small sample size, and the controls did not match the patients perfectly. Secondly, an inability to control for participants' thoughts during imaging is a limitation common to resting-state studies. Finally, small head movements and rotation are unavoidable even though participants were instructed not to move their heads and to rest with their eyes closed. However, we inspected each image, and patients with head movements greater than 2.5° or 2.5 cm were excluded. Given these limitations, these results should be considered preliminary and future studies should be well designed and should include larger numbers of participants.

## 5. Conclusions

In the present study, we examined changes in ALFF in LOD and NC groups at different frequency bands. The ALFF of primary effect of disease was widely distributed over left cerebellum anterior lobe, left cerebellum posterior lobe, left middle orbitofrontal gyrus, left superior occipital, and right superior parietal, while the interaction effect of disease and frequency was distributed over right superior frontal gyrus. This abnormal brain region of ALFF is related to total cognitive function and language function. So the widespread cortex anomaly may be depression-related character.

## Figures and Tables

**Table 1:** Demographic and neuropsychological data between LOD group and NC group.

Item	LOD group (*n* = 16)	NC group (*n* = 16)	*t*/*X* ^2^	*P* value
Age (years)	68.13 ± 5.24	68.25 ± 4.60	−0.072	0.943^a^
Gender (male : female)	8 : 8	8 : 8	0.000	1.000^b^
Education level (years)	10.88 ± 3.96	13.06 ± 2.74	−1.533	0.125^c^
HDRS	29.75 ± 5.07	1.94 ± 2.57	−4.858	0.000^c^
HARS	27.69 ± 7.12	1.81 ± 2.40	−4.809	0.000^c^
MMSE	28.81 ± 1.52	29.5 ± 0.82	−1.154	0.248^c^
AVLT-delayed recall	28.88 ± 4.80	36.31 ± 6.61	2.961	0.010^a^
Processing speed	0 ± 1.73	0 ± 1.79	0.006	0.995^a^
DST	10.88 ± 1.78	13.69 ± 1.78	−3.824	0.000^c^
VFT	24.81 ± 6.91	39.38 ± 7.64	4.870	0.000^a^
TMT-B	170.14 ± 59.04	152.11 ± 31.17	−1.144	0.270^a^

Note: LOD: late-onset depression; NC: normal controls; HDRS: Hamilton Depression Scale; HARS: Hamilton Anxiety Scale; MMSE: Mini Mental State Examination; AVLT-delayed recall: Auditory Verbal Learning Test-delayed recall; processing speed included the normalized symbol digit modalities test (SDMT) and Trail making test-A (TMT-A); DST: digit span test-forward and backward; VFT: verbal fluency test-animal and verb; TMT-B: trail making test-B.

^a^Two independent sample *t*-tests.

^b^Chi-square test.

^c^Mann-Whitney *U* test.

**Table 2:** The result of group × frequency ANOVA of ALFF.

Brain region	Peak MNI coordinates *x*, *y*, *z* (mm)	Peak value	Cluster size
(1) Main effect of group			
Cerebellum anterior lobe-L	−24, −39, −27	15.25	87
Cerebellum posterior lobe-L	−6, −66, −24	14.15	92
Frontal Mid Orb-L (aal)	−18,27, −15	20.14	80
Occipital Sup-L (aal)	−15, −84,45	22.05	89
Parietal Sup-R (aal)	24, −75,57	13.24	83
(2) Group × frequency interaction			
Superior frontal gyrus-R	39,54,3	6.2118	106

Note: a corrected threshold of *P* < 0.05 determined by Monte Carlo simulation was taken as meaning there was a significant difference. Cluster size is more than 2079 mm^3^. R = right; L = left; cluster size is in mm^3^; ANOVA = two-way analysis of variance.
